# Axillary lymph node dose with tangential whole breast radiation in the prone versus supine position: a dosimetric study

**DOI:** 10.1186/1748-717X-7-72

**Published:** 2012-05-18

**Authors:** Kara Lynne Leonard, David Solomon, Jaroslaw T Hepel, Jessica R Hiatt, David E Wazer, Thomas A DiPetrillo

**Affiliations:** 1 Department of Radiation Oncology, Tufts Medical Center, Box #593 800, Washington St, Boston, MA, 02111, USA; 2 Department of Radiation Oncology, Rhode Island Hospital, Warren Alpert School of Medicine of Brown University, Providence, RI, USA

**Keywords:** Breast cancer, Prone, Axillary lymph nodes, Radiation, ACOSOG Z0011

## Abstract

**Background:**

Prone breast positioning reduces skin reaction and heart and lung dose, but may also reduce radiation dose to axillary lymph nodes (ALNs).

**Methods:**

Women with early stage breast cancer treated with whole breast irradiation (WBI) in the prone position were identified. Patients treated in the supine position were matched for treating physician, laterality, and fractionation. Ipsilateral breast, tumor bed, and Level I, II, and III ALNs were contoured according to the RTOG breast atlas. Clips marking surgically removed sentinel lymph nodes (SLN)s were contoured. Treatment plans developed for each patient were retrospectively analyzed. V90_%_ and V95_%_ was calculated for each axillary level. When present, dose to axillary surgical clips was calculated.

**Results:**

Treatment plans for 46 women (23 prone and 23 supine) were reviewed. The mean V90_%_ and V95_%_ of ALN Level I was significantly lower for patients treated in the prone position (21% and 14%, respectively) than in the supine position (50% and 37%, respectively) (*p* < 0.0001 and *p* < 0.0001, respectively). Generally, Level II & III ALNs received little dose in either position. Sentinel node biopsy clips were all contained within axillary Level I. The mean V95_%_ of SLN clips was 47% for patients treated in the supine position and 0% for patients treated in the prone position (*p* < 0.0001). Mean V90_%_ to SLN clips was 96% for women treated in the supine position but only 13% for women treated in the prone position.

**Conclusions:**

Standard tangential breast irradiation in the prone position results in substantially reduced dose to the Level I axilla as compared with treatment in the supine position. For women in whom axillary coverage is indicated such as those with positive sentinel lymph node biopsy who do not undergo completion axillary dissection, treatment in the prone position may be inappropriate.

## Background

The eight-year results of the ACOSOG Z0011 study evaluating locoregional recurrence after sentinel lymph node dissection (SLND) with or without axillary lymph node dissection (ALND) in patients with positive sentinel lymph nodes suggest that completion ALND may be unnecessary for selected early stage breast cancer patients [1,2]. Patients treated on ACOSOG Z0011 received whole breast irradiation (WBI) with tangents in the supine position. Whole breast irradiation provides moderate radiation dose to the Level I and II axillary lymph nodes (ALN)s and to the region of the sentinel node [3-13]. If one considers that modest radiation doses to the axilla have clinical impact, the potentially practice-altering implications of ACOSOG Z0011 may reflect the importance of adequate dosing of these ALNs for sentinel node positive patients who have not had axillary dissection.

Recently, prone breast positioning for WBI has become more popular in efforts to decrease radiation dose to the heart and lungs and to decrease acute skin reaction in women with pendulous breasts [9,14-17] while maintaining acceptable long-term outcomes [18]. Treatment planning studies have demonstrated the dosimetric benefits of prone breast irradiation for all women, regardless of breast size [19]. Prone positioning increases the anatomic distance of the breast from the heart and lungs, eliminates the bolus effect created by the inframammary fold, and improves dose homogeneity as compared to WBI in the prone position. Recent work has shown decreased coverage of the ALNs in the prone position when treatment plans were created for each patient in both the prone and supine position [9]. The current work explores dosing to axillary lymph nodes in patients thought to be ideal candidates for prone positioning.

## Methods

Forty-six women with early stage breast cancer treated from 2009–2011 in the Departments of Radiation Oncology at our institution with standard tangential WBI in the prone or supine were identified. The 23 patients treated in the prone position were identified as ideal candidates for treatment in the prone position prior to or at the time of simulation for a variety of reasons including large breast size (*n* = 9), to limit lung dose (*n* = 11), or to limit heart dose (*n* = 3). Those treated prone for large breast size had a breast volume of 1000 mL or greater, those treated prone to limit lung dose would have had 2.5 cm or greater of cross-sectional lung included within a standard tangent, and those treated prone to limit heart dose had an anteriorly located left anterior descending (LAD) artery. Twenty-three patients treated in the supine position consecutively during the same time period (2009 – 2011) were chosen for comparison and matched for treating physician, laterality, and fractionation (conventional vs. Canadian fractionation [20]). All patients underwent Computed Tomography (CT) - based simulation using a Philips Brilliance Big Bore 16-slice CT scanner (Philips Healthcare, Andover, MA). Patients treated prone were simulated using the Bionix Prone Breast System (Bionix Radiation Therapy, Toledo, OH) and patients treated supine were simulated using the Accufix Quest Breast Board (Q-Fix Systems, Wyckoff, NJ). Three dimensional (3D) treatment planning for all patients was performed using the Pinnacle^3^ (version 8.0m, Philips Medical Systems, Cleveland, OH) treatment planning system. Conventional and prone breast tangential fields were designed to encompass the whole breast while minimizing dose to lung and heart without special attention to ALN coverage as confirmed by the treating physician. No patients were treated with “high tangents.”

**Figure 1:**
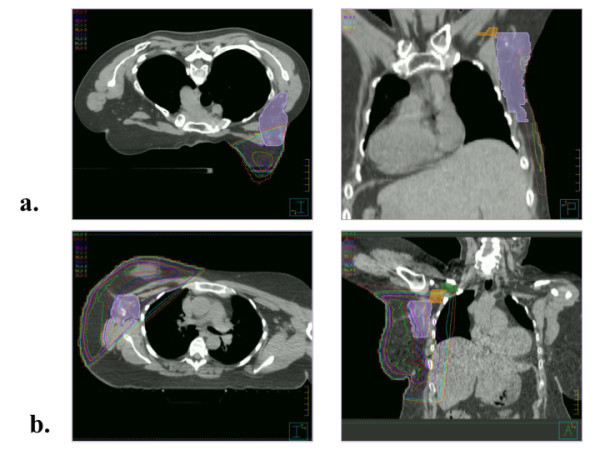
Axial and coronal images of Level I contoured (violet) for a patient treated in the (a) prone and (b) supine position.

Planning CT scans were retrospectively contoured to identify ipsilateral breast tissue, tumor bed, and Level I, II, and III ALNs according to the anatomic boundaries set by the Radiation Therapy Oncology Group (RTOG) contouring atlas [21], as shown in Figure 1. Table 1 delineates the boundaries of the levels in all 6 directions. When present, clips marking sentinel lymph nodes (SLN) removed during sentinel node biopsy were also contoured. Ipsilateral breast and ALN Level I-III volumes were compared between the two groups using the *t*-test.

**Table 1:** **Anatomic boundaries of the ipsilateral breast and axillary lymph node Levels I-III as defined by the Radiation Therapy Oncology Group (RTOG) contouring atlas **[1]

	**Cranial**	**Caudal**	**Medial**	**Lateral**	**Anterior**	**Posterior**
Breast	Clinical reference and second rib insertion	Clinical reference and loss of CT apparent breast	Sternal-rib junction	Clinical reference and mid axillary line. Excludes latissimus dorsi	Skin	Excludes pectoralis muscles, chestwall muscles, ribs
Level I	Axillary vessels crossing lateral edge of pectoralis minor	Pectoralis major insertion into ribs	Axillary vessels crossing lateral edge of pectoralis minor	Medial border of latissimus dorsi	Plane defined by the anterior surface of pectoralis major and latissimus dorsi	Anterior surface of subscapularis
Level II	Axillary vessels crossing medial edge of pectoralis minor	Axillary vessels crossing lateral edge of pectoralis minor	Medial border of pectoralis minor	Lateral border of pectoralis minor	Anterior surface of pectoralis minor	Ribs and intercostal muscles
Level III	Pectoralis minor insertion on corocoid process	Axillary vessels crossing medial edge of pectoralis minor	Thoracic inlet	Medial border of pectoralis minor	Posterior surface of pectoralis major	Ribs and intercostal muscles

Treatment plans for each patient were retrospectively analyzed to evaluate dose to the tumor bed, breast, and individual nodal levels. The mean dose to the ipsilateral breast as well as the volume of breast tissue and tumor bed receiving 95% (V95%) of the prescribed dose were calculated. The mean dose and the volume receiving 50% (V50%), 90% (V90%), and 95%, of the prescribed dose were then calculated for each axillary level. Dose to axillary surgical clips was similarly calculated, when applicable. Mean dose, V90%, V95% of axillary Level I were compared between those treated in the prone position and those treated in the supine position using a two-tailed unpaired *t*-test for difference of means. For Level I, the relative dose-volume histograms (DVH) were generated for all patients and the mean DVHs were compared among the prone and supine groups.

## Results

Table 2 provides patient characteristics for the prone and supine cohorts. In general, patients treated in the prone position were younger. The mean ipsilateral breast volumes and axillary Level I, II, and III nodal volumes (and standard deviations) are also shown in Table 2. As expected, breast volumes tended to be larger in the prone position than in the supine position, but this difference did not reach statistical significance. There was no difference in volumes contained within the axillary nodal levels between the two groups.

**Table 2:** Summary of patient characteristics

		**Supine (*n*= 23)**	**Prone (*n*= 23)**	**Comparison (two-tailed unpaired t-test)**
	Mean Age (y) ± SD*	65 ± 12	57 ± 9	
	Side: Left/Right	13/10	13/10	
	Mean breast volume (cm^3^) ± SD	841 ± 369	793 ± 462	*p* = 0.70 (CI^†^= -200 to 296)
				
T Stage	Tis	7	5	
	T1	12	17	
	T2	4	1	
N Stage	N0	21	21	
	N1	2	2	
Dissection Status:	No nodal biopsy	5	6	
	Sentinel node biopsy	18	15	
	Completion axillary dissection	0	2	
Radiation Dose	46 Gy in 23 fractions	8	8	
	42.5 Gy in 16 fractions	9	9	
	50-50.4 Gy in 25-28 fractions	6	6	
Mean Axillary Volume	Level I (cm^3^) ± SD	106 ± 38	98 ± 35	*p* = 0.46 (CI = -30 to 14)
	Level II (cm^3^) ± SD	21 ± 6	24 ± 7	
	Level III (cm^3^) ± SD	9 ± 3	10 ± 5	*p* = 0.13 (CI = -0.87 to 6.9)
				
				*p* = 0.42 (CI = -3.5 to 1.5)

* *SD* = standard deviation; ^†^*CI* = Confidence interval.

Doses (and standard deviations) delivered to the ipsilateral breast, tumor bed, and all axillary levels are provided in Table 3a. Mean breast dose and V95% of ipsilateral breast and tumor bed did not differ significantly between those treated in the prone position and those treated in the supine position. Only axillary Level I received appreciable dose in both groups of patients. Generally, Level II & III ALNs did not receive a significant portion of prescribed dose in either position.

**Table 3:** Summary of calculated volume and dose to (a) ipsilateral breast and tumor bed, to (b) axillary levels I, II, and III and to (c)sentinel lymph node (SLN) clips

**a) Dose to ipsilateral breast and tumor bed**				
		**Supine (*n* = 23)**	**Prone (*n*= 23)**	**Comparison (two-tailed unpaired t-test)**
Ipsilateral breast mean dose (%) ± SD*		96 ± 3.8	95 ± 1.8	*p* = 0.26 (CI^††^ = -0.77 to2.8)
				
V95_%_ ipsilateral breast		77 ± 6.5	74 ± 5.3	*p* = 0.09 (CI = -0.52 to 6.5)
				
V95_%_ tumor bed		99 ± 1.7	98 ± 4.6	*p* = 0.33 (CI = -1.1 to 3.1)
**b) Dose to axillary volumes**				
Mean dose (%) ± SD	Level I	66 ± 20	36 ± 30	*p*< 0.0001 (CI =15 to 45)
	Level II	6 ±15	3 ± 3	
	Level III	3 ± 2	< 1 ± 0.4	
Mean V95_%_ ± SD	Level I	37 ± 18	14 ± 19	*p* < 0.0001 (CI = 12 to 34)
	Level II	0	0	
	Level III	0	0	
Mean V90_%_ ± SD	Level I	50 ± 20	21 ± 25	*p*< 0.0001 (CI = 16 to 43)
	Level II	1 ± 5	0	
	Level III	0	0	
Mean V50_%_ ± SD	Level I	67 ± 19	35 ± 33	
	Level II	5 ± 17	< 1 ± 2	
	Level III	0	0	
**c) Dose to sentinel lymph node (SLN**^**†**^**) clips**				
		**Supine (*n* = 9)**	**Prone (*n* = 7)**	
SNB clips in treatment field		9	2	
V95_%_ SLN clips ± SD		47 ± 43	0 ± 0	*p*<0.0001 (95% CI =29 to 65)
SLN clips receiving > 90% dose		8	1	
Mean dose to SLN clips (%) ± SD		95 ± 5	43 ± 34	

*SD = standard deviation; ^†^*SLN* = sentinel lymph node; ^††^*CI* = confidence interval.

**Figure 2:**
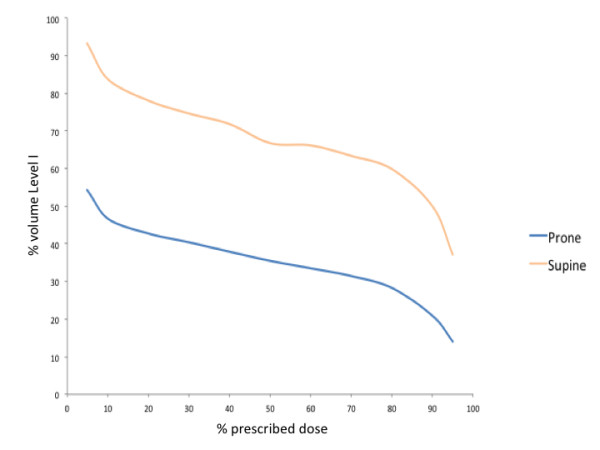
Comparison of mean DVH data for patients treated in the prone and supine position.

Within each group, there was substantial variation in the dose coverage of axillary Level I. Among women treated in the prone position, V95_%_ was less than 10% in 14 women and was 0% in 6 women. Figure 2 compares the mean Level I DVH for the prone and supine groups. The mean dose to Level I was 36% of prescription for patients treated in the prone position, significantly less than the 66% of prescription for patients treated in the supine position (*p* < 0.0001; 95% confidence interval = 15% to 45%). Mean V95_%_ was lower in women treated in the prone position as compared to women treated in the supine position (14% vs. 37%; *p* < 0.0001; 95% confidence interval = 12% to 34%).

Sixteen patients (seven treated prone and nine treated supine) had axillary surgical clips placed at the time of SLN biopsy; all of the clips were located within axillary Level I. Only two of the seven patients treated in the prone position had clips within the tangential treatment field. However, all SLN clips in patients treated in the supine position were within the treatment field. As shown in Table 3c, mean V95% of SLN clips was 0% for the seven patients treated in the prone position. Mean V95_%_ of SLN clips was 47% among patients treated in the supine position. The difference in mean V95% between the two groups was statistically significant (*p* < 0.0001; 95% confidence interval = 29% to 65%). At least 90% of prescribed dose was delivered to the clips in eight of nine patients treated supine (mean V90_%_ = 96%), but in only one of seven patients treated prone (mean V90% = 13%).

## Discussion

The recently published eight-year results of the American College of Surgeons Oncology Group (ACOSOG) Z0011 study showed no difference in axillary failure rates or overall survival in women with cT1-2 cN0 breast cancer with one or two positive sentinel nodes randomized to undergo no further axillary surgery versus completion axillary dissection [1,2]. The locoregional control was excellent (1.8% failure) despite suspected residual disease in approximately 40% of these women. Per protocol, women in this study received adjuvant radiation therapy with standard tangential irradiation. Although there has been debate surrounding the contribution of radiation therapy to axillary control in these women, this was likely due to sterilization of micrometastatic disease by tangential radiation therapy. These findings have strong implications for the importance of adjuvant radiation therapy to provide dose to the lower axilla in women with positive sentinel lymph nodes who will not undergo completion axillary dissection. In fact, regional nodal irradiation has also been associated with improved survival [22], and improvements in locoregional control have translated into improvements in overall survival with extended follow-up [23].

Prone breast irradiation provides clear benefits in reducing skin toxicity for large breasted women and in reducing heart and ipsilateral lung dose for patients with unfavorable thoracic anatomy [9,14-18]. The present investigation suggests that axillary nodal coverage should also be considered when making the decision to treat women in the prone versus supine position. The data presented in this study suggest that irradiation in the prone position does not allow for the same coverage of axillary tissue as does irradiation in the supine position.

Although the classic anatomic boundaries of the axilla are well defined, the clinically relevant borders are not. There is substantial variation in definition of boundaries of the axilla used in radiotherapy treatment as outlined in Table 4. For the purpose of standardizing the borders of the axilla, the RTOG published a contouring atlas [21]. Perhaps contrary to classic teaching, recent literature (including the present study) suggests that standard tangential whole breast irradiation delivers the prescribed dose to only a portion of Level I of the axilla, particularly as defined by the RTOG. In the present study, only 37% of the Level I volume received 95% of the dose with standard tangential irradiation. It is not clear whether this level of coverage delivers sufficient dose for therapeutic treatment of axillary lymph nodes. Studies demonstrating similar coverage of axillary Level I with standard tangents have suggested that such coverage does not provide adequate doses to control residual microscopic disease in the axilla [3,7]. The present study suggests that the area of the axilla receiving fully dose (90-95% of the prescribed dose) may contain the microscopic disease. In fact, the clinically important target tissue in the axilla has not yet been clearly defined. The 98% locoregional control seen in ACOSOG Z0011 with SNLD alone suggests that standard tangential fields encompass the clinically relevant nodal tissue.

**Table 4:** Review of the literature reporting boundaries of and dose to axillary Level I volumes and/or clips in patients undergoing tangential radiation therapy as part of breast conservation therapy

**Study**	**Patients (*n*)**	**Method**	**Axillary surgery**	**Axillary clips (*n)***	**Borders of axillary Level I**	**Dose to Level I**
Krasin *et al.*[3]	25	2D sim/3D analysis	ALND*	16	Not listed	Mean dose = 32 Gy (63.5%)
Aristei *et al.*[4]	35	2D sim/3D analysis	ALND	35	Caudal border: Inferior clip	D_90_% = 6.75 Gy
					Cranial border: Axillary vein and 2^nd^ clip	
					Medial border: Lateral border of pectoralis minor	
					Lateral border: Axillary vein	
					Posterior border: Latissimus dorsi	
Takeda *et al.*[5]	44	2D sim/3D analysis	ALND	44	Caudal border: Clip in latissimus dorsi at inferior level of dissection	Median V_80_% = 30.5%
					Cranial border: Clip in the latissimus dorsi at level of axillary vein	
					Posterior border: Clip in adjacent to subscapularis vein	
Orecchia *et al.*[6]	15	3D CRT^†^	SNB^††^	15	Caudal border: SNB clip	Mean dose = 48.7%
					Cranial border Manubrium	± 22%
	35	3D CRT	SNB	N/A	Caudal border: Between 4^th^ and 5^th^ ribs	V_95_% = 51% ± 16%
Reznik *et al.*[7]					Cranial border: Axillary vein	Mean dose = 66% ± 13%
					Medial border: Pectoralis minor	
Reed *et al.*[8]	50	3D CRT	32 SNB	50	Caudal border: Latissiumus dorsi and clavipectoral-lattisimus fascia	Mean V_95_% = 55% Median V_95_% = 53%
			18 ALND		Cranial border: Most inferior axial image of axillary artery/vein	
					Medial border: Pectoralis minor	
					Lateral border: Medial aspect of pectoralis seratus	
					Anterior border: Pectoralis minor	
					Posterior border: Latissimus dorsi and subscapularis	
Alonso-Basanta *et al.*[9]	20	3D CRT	Not reported	N/A	Caudal border: Origin of pectoralis minor 3^rd^ – 5^th^ ribs	V_95_% < 60%
					Cranial border: Pectoralis minor insertion of the coracoid process	Prone: Mean dose = 11.2 Gy
					Medial border: Pectoralis minor	Supine: Mean dose = 21 Gy
					Lateral border: Latissimus dorsi	
					Posterior border: Latissimus dorsi	
Schlembach *et al.*[10]	105	3D CRT	65 SNB	105	Surgical clips	85% of clips in the field
						Mean dose to clips = 98%
			39 ALND			
McCormick *et al.*[11]	45	2D	ALND	45	Surgical clips	38% of clips in field
Chung *et al.*[12]	36	2D or 3D CRT	SNB	36	Surgical clips	94% of clips in field
Present study	46	3D CRT	SNB	7 prone	RTOG Atlas [20]	Prone: V_95_% = 14%
				9 supine		Mean dose = 36% ± 31%
						Mean dose to clips = 43%
						Supine: V_95_% = 37% (supine)
						Mean dose = 66% ± 20%
						Mean dose to clips = 95%

*ALND = Axillary lymph node dissection; ^†^*3DCRT* = Three dimensional conformal radiation therapy; ^††^*SNB* = Sentinel node biopsy.

The location of clips placed during SNLD may represent the area at highest risk of harboring metastatic disease after SNLD alone. Certainly, we know that the sentinel node is the most likely to contain breast cancer metastases. The neighboring nodes may represent the next echelon for metastatic spread. Despite suboptimal coverage of the entire nodal volume, standard tangents provide substantial dose to the clips placed to mark the location of the sentinel lymph node as shown in this study as well as others [8,10,12]. However, for women treated in the prone position, the area marked by SNLD clips receives minimal radiation dose. This raises concern that treatment in the prone position may provide suboptimal coverage of the most clinically important axillary nodal areas.

The study by Stegman et al., which showed a 5-year regional control rate of 98.4% in 245 patients treated with WBI in the prone position demonstrates that prone irradiation provides adequate adjuvant treatment for the pathologically negative axilla and for the positive axilla that has undergone a full Level 1, 2 axillary lymph node dissection [18]. This excellent axillary control rate is expected in a population of women comprised of 15% of patients with DCIS and 65% of patients with pN0 disease. Importantly, all women with positive lymph nodes in the Stegman study underwent Level 1, 2 axillary lymph node dissection. The undissected node-positive population in the ACOSOG Z0011 is at substantially higher risk for regional failure and likely receives substantial benefit from standard tangential irradiation.

Results similar to ours were published by Alonso-Basanta et al., who reported on twenty patients simulated in both the prone and supine position [9]. The mean dose to the axilla was 11.2 Gy when patients were simulated in the prone position and 21 Gy when the same patients were simulated in the supine position. The authors stated that alternative positioning was warranted in eight of the twenty patients for anatomic reasons (i.e., breast size, lung dose), while in the present study, all twenty-three patients treated prone were determined to be ideal candidates for prone positioning by the treating radiation oncologist. Thus, the current investigation includes patients in whom such a dosimetric evaluation is most critical. Moreover, the present study includes the novel finding that tangential irradiation in the supine position provides significantly better coverage of sentinel lymph node tissue than tangential irradiation in the prone position.

The limitations of the present study include a small sample size and the inherent sample bias associated with retrospective trial design. Additionally, only a small number of patients had SNLD clips placed, limiting the analysis of radiation dose delivery to axillary clips. While care was taken to choose an appropriate supine match for each prone patient, differences may inherently exist between the two groups. Although there was no significant difference in mean breast size or mean axillary volume between the two groups, other differences may exist that could bias the results. For example, patients in the prone group are slightly younger than patients in the supine group.

## Conclusion

Standard tangential breast irradiation in the prone position results in substantially reduced dose to the Level I axilla as compared with treatment in the supine position. For women in whom axillary coverage is indicated such as those with positive sentinel lymph node biopsy who do not undergo completion axillary dissection, treatment in the prone position may be inappropriate. For women with DCIS or those with a negative sentinel node biopsy, treatment in the prone position does result in good coverage of target breast tissue.

## Abbreviations

ALN, Axillary lymph node; WBI, Whole breast irradiation; SLN, Sentinel lymph node; SLND, Sentinel lymph node dissection; ALND, Axillary lymph node dissection; CT, Commuted tomography; RTOG, Radiation Therapy Oncology Group; V90_%_, Volume receiving 90% of the dose; V50_%_, Volume receiving 50% of the dose; V95_%_, Volume receiving 95% of the dose; DVH, Dose volume histogram.

## Competing interests

The authors declare that they have no competing interests.

## Authors’ contributions

KLL performed data collection, analysis and interpretation of data, was involved in drafting the manuscript and revising it critically for important intellectual content. DS performed data collection, analysis and interpretation of data, was involved in drafting the manuscript. JTH was involved in drafting the manuscript and revising it critically for important intellectual content. JRH was involved in analysis and interpretation of data. DEW made substantial contributions to conception and design of the study and revised in critically for important intellectual content. TAD was principally responsible for the conception and design of the study and revised in critically for important intellectual content. All authors read and approved the final manuscript.
